# Supplementary material for: Reconciling Mining with the Conservation of Cave Biodiversity: A Quantitative Baseline to Help Establish Conservation Priorities
Source: PLoS One. 2016 Dec 20;11(12):e0168348. doi: 10.1371/journal.pone.0168348 (PMC5173368; doi:10.1371/journal.pone.0168348)
Supplement: S1 Dataset — (ZIP) [file pone.0168348.s002.zip › Taxa/Serra Norte/SN_2013/N5S/Lista N5S-15.pdf]

CAVIDADE N5S-0015

Classe	Ordem	Fam/Outros	Gên/Outros	Espécie	S-U	U-S	Zon.
Arachnida	Amblypygi	Phrynidae	*Heterophrynus*	*longicornis*	1	X	
Arachnida	Araneae	Barychelidae		jovem	1		A
Arachnida	Araneae	Corinnidae		jovem	1		P
Arachnida	Araneae	Filistatidae		sp.1	X	X	
Arachnida	Araneae	Ochyroceratidae	*Ochyrocera*	sp.1	X		E
Arachnida	Araneae	Oonopidae	aff. *Triaeris*	sp.2	X		P
Arachnida	Araneae	Pholcidae	*Leptopholcus*	sp.1	X		P
Arachnida	Araneae	Pholcidae	*Mesabolivar*	sp.1	X		A
Arachnida	Araneae	Pholcidae		jovem		X	
Arachnida	Araneae	Salticidae		sp.7	X		E
Arachnida	Araneae	Scytodidae	*Scytodes*	*itapevi*		1	
Arachnida	Araneae	Scytodidae	*Scytodes*	sp.1	12		E/P
Arachnida	Araneae	Tetragnathidae	*Azilia*	sp.		X	
Insecta	Blattodea			jovem	1		E
Insecta	Coleoptera	Anthicidae		sp.1	X		E
Insecta	Coleoptera	Chrysomelidae		sp.2	X		E
Insecta	Coleoptera	Dermestidae		jovem		X	
Insecta	Coleoptera	Dytiscidae		sp.11C	X		E
Insecta	Diptera	Chloropidae		sp.	X		E
Insecta	Diptera	Drosophilidae		jovem		X	
Insecta	Diptera	Milichiidae	*Milichiella*	sp.	X		P
Insecta	Diptera	Muscidae		sp.	X		P
Insecta	Diptera	Psychodidae	Phlebotominae	sp.	X		E
Insecta	Heteroptera	Reduviidae	Emesinae	sp.		X	
Insecta	Heteroptera	Reduviidae	Reduviinae	sp.	3	X	E/P/A
Insecta	Heteroptera	Reduviidae	Triatominae	jovem	1		P
Insecta	Heteroptera	Veliidae	*Paravelia*	jovem	X		E
Insecta	Hymenoptera	Bethylidae		sp.1	X		P
Insecta	Hymenoptera	Braconidae		sp.1	X		P
Insecta	Hymenoptera	Formicidae	*Camponotus*	sp.2	X	X	E/P
Insecta	Hymenoptera	Formicidae	*Dolichoderus*	aff. *bispinosus*	X		E
Insecta	Hymenoptera	Formicidae	*Gnamptogenys*	sp.1	X		P
Insecta	Hymenoptera	Formicidae	*Labidus*	sp.1	X		E
Insecta	Hymenoptera	Formicidae	*Platythyrea*	sp.1		X	
Insecta	Hymenoptera	Formicidae	*Solenopsis*	sp.		X	
Insecta	Hymenoptera	Formicidae	*Solenopsis*	sp.1	X		P
Insecta	Hymenoptera	Formicidae	*Solenopsis*	sp.2	X		P
Insecta	Isoptera	Termitidae	*Nasutitermes*	sp.	X	X	E/P
Insecta	Lepidoptera	Noctuoidea		sp.5	1		A
Insecta	Lepidoptera	Tineoidea		sp.		X	
Insecta	Lepidoptera	Tineoidea		sp.1	X		P
Insecta	Lepidoptera	Tineoidea		sp.2	X		E/P
Insecta	Lepidoptera	Tineoidea		sp.3	X		P
Insecta	Neuroptera	Myrmeleontidae		jovem	X		E/P
Insecta	Orthoptera	Phalangopsidae	*Paraclodes*	jovem	3	X	A
Insecta	Orthoptera	Phalangopsidae	*Phalangopsis*	sp.	33	X	A
Insecta	Psocoptera	Psyllipsocidae	*Psocathropos*	sp.1	X		P
Insecta	Psocoptera	Psyllipsocidae	*Psyllipsocus*	sp.3	X		P
Gastropoda	Pulmonata	Systrophiidae	*Happia*	sp.		X	

Malacostraca	Isopoda	Philosciidae		sp.2	X		E
Amphibia	Anura	Strabomantidae	*Pristimantis*	cf. *fenestratus*	1		E
Mammalia	Chiroptera	Emballonuridae	*Peropteryx*	*kappleri*	10	X	P
Mammalia	Chiroptera	Phyllostomidae	*Diphylla*	*ecaudata*	1		P
Mammalia	Chiroptera	Phyllostomidae	*Trachops*	*cirrhosus*	6	X	P
Mammalia	Rodentia	Cricetidae	*Rhipidomys*	sp.		1	
Reptilia	Squamata	Gekkonidae	*Thecadactylus*	sp.	1		P
